# High-frequency pseudo-hypofunction of the superior semicircular canal dehiscence syndrome: a vHIT – oVEMP dissociation explained by hydrodynamic energy loss

**DOI:** 10.1007/s00405-026-10347-0

**Published:** 2026-06-01

**Authors:** Leonardo Manzari, Nicola Ferri, Marco Tramontano

**Affiliations:** 1 MSA ENT Academy Center, Cassino (FR), Italy; 2 Translational Rehabilitation Sciences Group, Department of Biomedical and Neuromotor Sciences (DIBINEM), Alma Mater Studiorum University of Bologna, Bologna, 40138 Italy; 3 Unit of Occupational Medicine, IRCCS Azienda Ospedaliero-Universitaria di Bologna, Bologna, 40138 Italy

**Keywords:** Semicircular canal dehiscence, Video head impulse test, kHz vestibular evoked myogenic potentials, Vestibular hair cells, Third window syndrome, Transient vestibular system

## Abstract

**Background:**

Superior semicircular canal dehiscence syndrome (SCDS) produces a wide spectrum of vestibular and auditory abnormalities due to the presence of a “third mobile window.” While enhanced otolith-mediated responses are well recognized, the behavior of high-frequency semicircular canal function remains debated. Many patients show reduced superior canal vestibulo-oculomotor reflex (VOR) gains despite preserved or exaggerated utricular responses.

**Objective:**

To investigate the relationship between dynamic semicircular canal responses assessed by video Head Impulse Test (vHIT) and otolith responses assessed by oVEMPs in people with superior semicircular canal dehiscence.

**Methods:**

Fifty-three patients with SCDS underwent full vestibular testing including vHIT of all six semicircular canals, cervical and ocular vestibular-evoked myogenic potentials (cVEMPs, oVEMPs) to 500-Hz BCV, and high-frequency oVEMPs to 4,000-Hz stimuli.

**Results:**

Superior semicircular canals showed the largest VOR gain reductions, with the left anterior (LA) canal most affected (mean gain 0.86 ± 0.18). Bilateral SCDS exhibited significantly lower gains than unilateral SCDS, with mean LA gain falling below the functional threshold (0.68 ± 0.14). In contrast, all patients demonstrated enhanced utricular responsiveness, including increased 500-Hz oVEMP amplitudes and consistent 4-kHz N10 responses.

**Conclusion:**

Our findings confirm the presence of high-frequency oVEMP hyperresponsiveness, yet with a VOR pseudo-hypofunction due to a loss of high-frequency mechanical energy through the dehiscence, which diminishes the utricular vortex and cupular deformation. This frequency-dependent hydrodynamic dissociation provides a comprehensive mechanistic framework for interpreting vestibular test batteries in SCDS. Combined assessment with vHIT and high-frequency oVEMPs offers a powerful, physiologically grounded diagnostic approach.

## Introduction

Superior semicircular canal dehiscence syndrome (SCDS) is characterized by the presence of an abnormal third window in the bony labyrinth, which fundamentally alters the transmission of acoustic, pressure, and inertial stimuli within the inner ear. Patients typically exhibit a constellation of auditory and vestibular symptoms, like autophony, low-frequency air–bone gaps, sound- or pressure-induced vertigo (Tullio and Hennebert phenomena), and hypersensitivity to internal body sounds, reflecting the complex hydrodynamic consequences of the dehiscence [1]. Over the last decade, advances in vestibular physiology have shown that SCDS does not simply augment otolithic responses, but also produces distinctive effects on semicircular canal function, particularly during high-frequency stimulation [2].

The video Head Impulse Test (vHIT) provides a measure of semicircular canal function under high-acceleration, transient conditions, stimulating Type I irregular afferents located at the center of the ampullary crest [3, 4]. Paradoxically, several studies have reported that many patients with SCDS exhibit reduced vHIT gains of the superior semicircular canal (SSC), despite preserved or exaggerated low-frequency vestibular-evoked myogenic potentials (VEMPs). Mukherjee and colleagues [5] first emphasized this dissociation, while Tikka et al. highlighted the variability of vHIT responses and suggested that altered canal mechanics may underlie the phenomenon [6]. Castellucci and colleagues further demonstrated that SSC vHIT gain correlates with dehiscence size and location, suggesting a biomechanical rather than neurophysiological mechanism of dysfunction [7]. Concurrently, high-frequency ocular VEMPs (oVEMPs), and particularly the 4-kHz N10 response, have emerged as the most specific and sensitive functional marker of SCDS. A previous study [2] demonstrated that the presence of a 4-kHz oVEMP has 100% sensitivity and 100% specificity for SCD, a finding subsequently confirmed and physiologically explained by Manzari and colleagues [8]. Their neurophysiological recordings showed that after a dehiscence is created, both utricular Type I afferents and superior canal afferents become abnormally responsive to high-frequency sound, providing a direct neural substrate for the exaggerated oVEMP response. The presence of robust 4-kHz oVEMPs therefore constitutes strong evidence that Type I hair cells remain intact and hyperresponsive, ruling out sensory loss as the cause of reduced vHIT gains.

A major advance in understanding the apparent vHIT–oVEMP paradox comes from the fluid–structure interaction (FSI) model developed by Goyens et al. [9]. Their simulations revealed that high-frequency head impulses generate a vortical flow of endolymph in the utricle, producing a characteristic asymmetric, S-shaped cupular deformation that selectively engages Type I receptors. This model demonstrates that efficient high-acceleration vestibulo-ocular reflex (VOR) responses depend critically on the integrity of canal hydrodynamics. In the presence of a dehiscence, a portion of this mechanical energy is lost through the third window, attenuating the cupular deformation required for high-frequency VOR generation [10]. Thus, reduced vHIT gain reflects hydrodynamic energy loss, not neuroepithelial injury. At the same time, the third window enhances the transmission of pressure and acoustic stimuli, explaining the exaggerated oVEMP responses seen in SCDS, including the universally present 4-kHz responses.

Taken together, the emerging view is that SCDS produces a frequency-dependent hydrodynamic dissociation, in which high-frequency rotational stimuli (vHIT) are dampened, while high-frequency acoustic stimuli (oVEMP) are amplified. This dissociation represents a form of high-frequency pseudo-hypofunction of the SSC. However, data from larger cohorts integrating both measures are limited, and the pathophysiological basis of this dissociation remains insufficiently defined.

Recent biomechanical models of third-window lesions have suggested that the presence of a dehiscence may alter the hydrodynamic impedance of the labyrinth and redistribute mechanical energy within the inner ear system [2]. Within this framework, part of the stimulus energy may be dissipated through the dehiscent canal, potentially modifying semicircular canal dynamics while simultaneously enhancing vestibular sensitivity to sound and vibration. The present study was therefore designed to explore whether such differential effects could be observed clinically as a dissociation between dynamic semicircular canal responses assessed by vHIT and otolith responses assessed by oVEMPs in patients with SCDS.

In the light of above, this study aims to investigate the relationship between dynamic semicircular canal responses assessed by vHIT and otolith responses assessed by oVEMPs in people with superior semicircular canal dehiscence. By interpreting the results in the context of current biomechanical and physiological models, we aimed to clarify the mechanisms underlying the divergent effects of SCDS on canal and otolith pathways.

## Methods

This retrospective observational study was conducted according to the Strengthening the Reporting of Observational Studies in Epidemiology (STROBE) guidelines [11]. The analysis was performed on vestibular data previously collected during routine clinical assessments at our institution. All procedures contributing to this work comply with the ethical standards of the relevant national and institutional guidelines on human experimentation and with the World Medical Association Declaration of Helsinki. Consecutive adult patients evaluated at the MSA ENT Center between 2015 and 2024 were considered for inclusion. Patients were included if they met all the following criteria: age ≥ 18 years at the time of evaluation; referral for vestibular and/or auditory symptoms compatible with a third-window lesion (e.g. sound- or pressure-induced vertigo or oscillopsia, autophony, hyperacusis to bone-conducted sounds, aural fullness, conductive-like hearing loss with normal middle ear status); availability of a complete vestibular test battery, including vHIT of all six semicircular canals, and ocular and/or cervical VEMPs to bone-conducted vibration (BCV) and/or air-conducted sound (ACS) performed according to the standardized protocol described in this study; stable clinical condition and ability to cooperate with vestibular testing and to provide informed consent. Patients were excluded if one or more of the following applied: clinically relevant middle ear disease that could bias VEMPs or vHIT results (e.g. chronic otitis media, cholesteatoma, ossicular chain discontinuity, otosclerosis, or persistent middle ear effusion/marked negative middle ear pressure at the time of testing); previous otologic or neuro-otologic surgery involving the labyrinth, middle ear, or posterior fossa, including prior SSCD plugging or resurfacing; central nervous system disorders potentially affecting the vestibulo-ocular reflex or ocular motor function (e.g. multiple sclerosis, cerebellar degeneration, brainstem or cerebellar stroke, relevant neurodegenerative disease); recent vestibular insults or vestibulotoxic exposure, such as acute peripheral vestibulopathy (vestibular neuritis, labyrinthitis) or documented exposure to vestibulotoxic drugs within 6 months prior to testing; incomplete or non-interpretable vestibular recordings, including poor fixation or goggle slippage preventing reliable VOR gain calculation on vHIT, or artefact-contaminated, irreproducible, or absent VEMP waveforms precluding confident interpretation. All participants gave written consent to publish the results obtained from their clinical examinations and instrumental tests.

### The vestibular assessment

The function of the semicircular canals was measured using vHIT (OtosuiteV^®^, GN Otometrics, Denmark). Gain value < 0.76 for horizontal semicircular canals and < 0.66 for the vertical semicircular canals identified the hypofunction of VOR [12]. Head impulses were delivered unpredictably in the planes of the semicircular canals with peak head velocities typically ranging between 150 and 250°/s, consistent with recommended clinical vHIT protocols. At least 15 valid impulses were delivered for each canal to ensure reliable gain estimation. Both overt and covert corrective saccades were inspected during analysis of the vHIT traces. The otolith function was evaluated with ocular VEMPs. For the utricular macula was considered the n10 (5–10 µV) negative (excitatory), crossed, the vestibular-evoked myogenic potential of the stretched inferior oblique eye muscles recorded by surface EMG electrodes on the skin beneath the eyes in response to stimulation by BCV delivered to the midline of the forehead at the hairline (Fz), or by ACS. Based on the evidence of utricular–ocular projections and neural evidence of the preferential activation of irregular otolithic afferent neurons by 500 Hz BCV and ACS [2], the oVEMP (n10) to these stimuli is held to index mainly utricular function.

For oVEMPs, active electrodes were placed approximately 1 cm below each eye, reference electrodes approximately 1–2 cm inferior to the active electrode, and the ground electrode on the forehead. Participants were instructed to maintain an upward gaze during recording to activate the inferior oblique muscles. For cVEMPs, electrodes were placed over the middle portion of the sternocleidomastoid muscle with the reference electrode over the sternum. Participants were asked to maintain tonic contraction of the sternocleidomastoid muscle.

### Statistical analysis

The Asymmetry Ratio (AR) between the affected and healthy sides was calculated, with values ≥ 40 considered pathological. For each semicircular canal, the average horizontal and vertical slow-phase VOR gain was obtained by summing the gain values across all repetitions. All statistical analyses were conducted using STATA 19.5 software (StataCorp, 2025; College Station, TX, USA). Continuous variables are reported as mean ± standard deviation or median with interquartile range, depending on their distribution, whereas categorical variables are presented as counts and percentages. The normality distribution was assessed using the Shapiro–Wilk test. For comparisons between groups defined by the affected side (left, right, or bilateral), one-way ANOVA was applied to normally distributed variables, while the Kruskal–Wallis test was used for non-normally distributed variables. The prevalence of dysfunctional canals (categorized as hypofunction, normal function, or hyperfunction) was compared across groups using χ² tests or Fisher’s exact test when appropriate. All tests were two-tailed, and statistical significance was set at *p* < 0.05.

## Results

Fifty-three patients with complete vHIT assessment and TC-confirmed SCDS were included in the analysis, clinical and demographic characteristics are reported in Table 1. Twenty-six individuals presented left-sided SCDS (49.06%), 17 right-sided SCDS (32.08%), and 10 exhibited bilateral involvement (18.87%).

**Table 1:** Sample characteristics (*n* = 53)

Age, years (mean ± SD)	51.8 ± 15.2
Sex, female (%)	23 (43.4)
Affected side	
Left	26 (49.0)
Right	17 (32.1)
Bilateral	10 (18.9)

All canals except the left anterior (LA) demonstrated non-normal distributions (*p* < 0.05), indicating significant skewness and variability in canal performance (Table 2).

**Table 2:** Shapiro-Wilk test for VOR gains

	W	*P*
Right lateral	0.878	< 0.001
Left lateral	0.913	0.001
Right anterior	0.951	0.031
Right posterior	0.934	0.006
Left posterior	0.827	< 0.001
Left anterior	0.973	0.274

Each semicircular canal presents an average VOR gain above the functional threshold. The median or mean VOR gains for each semicircular canal are summarized in Table 3.

**Table 3:** VOR gains for each semicircular canal

	VOR gain (median and IQR)
Right lateral	1.02 (0.98, 1.09)
Left lateral	1.01 (0.97, 1.07)
Right anterior	0.91 (0.85, 1.00)
Left anterior	0.86 ± 0.18*
Right posterior	0.89 (0.84, 0.95)
Left posterior	0.89 (0.84, 0.95)

*: mean and SD

**Fig. 1:**
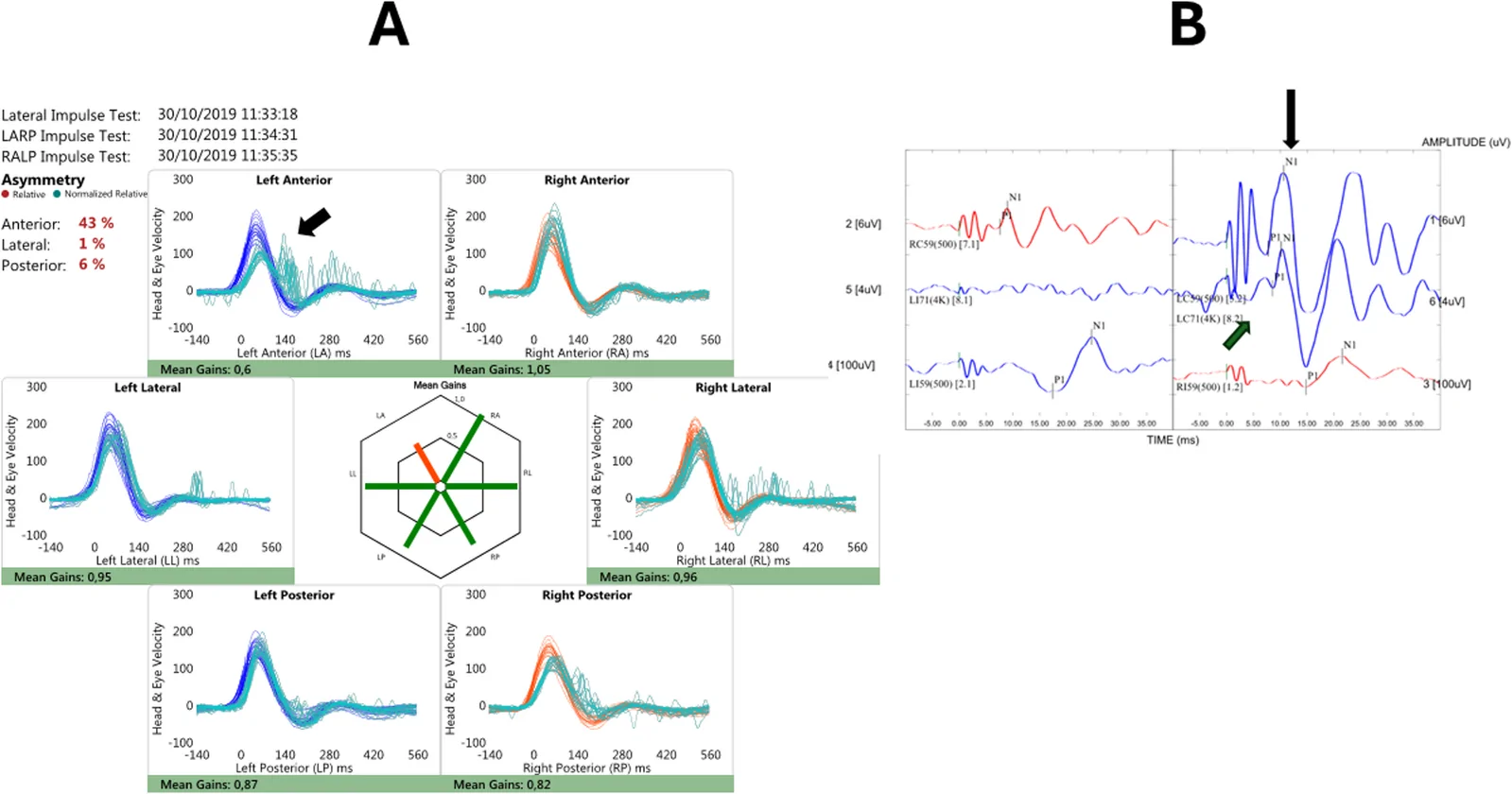
Pseudo-hypofunction on vHIT with enhanced BCV o/cVEMPs in left superior semicircular canal dehiscence, vHIT and bone-conducted vibration (BCV) VEMPs in a patient with left superior semicircular canal dehiscence (SSCD). (**A**) video Head Impulse Test (vHIT) recorded at the MSA ENT Academy Center, Cassino (Italy), showing selective reduction of the vestibulo-ocular reflex (VOR) gain for rapid, unpredictable head impulses in the plane of the left anterior (superior) semicircular canal (mean gain 0.6; black arrow), with preserved gains in the remaining canals. This pattern is consistent with an apparent canal hypofunction (pseudo-hypofunction) associated with SSCD. (**B**) Raw traces of ocular and cervical VEMPs evoked in the same subject by BCV delivered at Fz (tone burst: 0 ms rise time, 1 ms plateau) at 500 Hz (upper rows) and 4000 Hz (middle rows); the lower rows show cervical VEMPs recorded from the sternocleidomastoid muscles. On the left of panel B are responses from the left eye and left sternocleidomastoid muscle, and on the right those from the right side. A large n10 potential is clearly identifiable beneath the eye contralateral to the affected ear at 4000 Hz (black arrow), together with increased BCV-VEMP amplitudes (green arrow), indicating hypersensitivity of otolithic/superior canal afferents. The figure illustrates the characteristic dissociation in SSCD between pseudo-hypofunction of the affected superior canal on vHIT and enhanced, high-frequency BCV-VEMP responses.

**Fig. 2:**
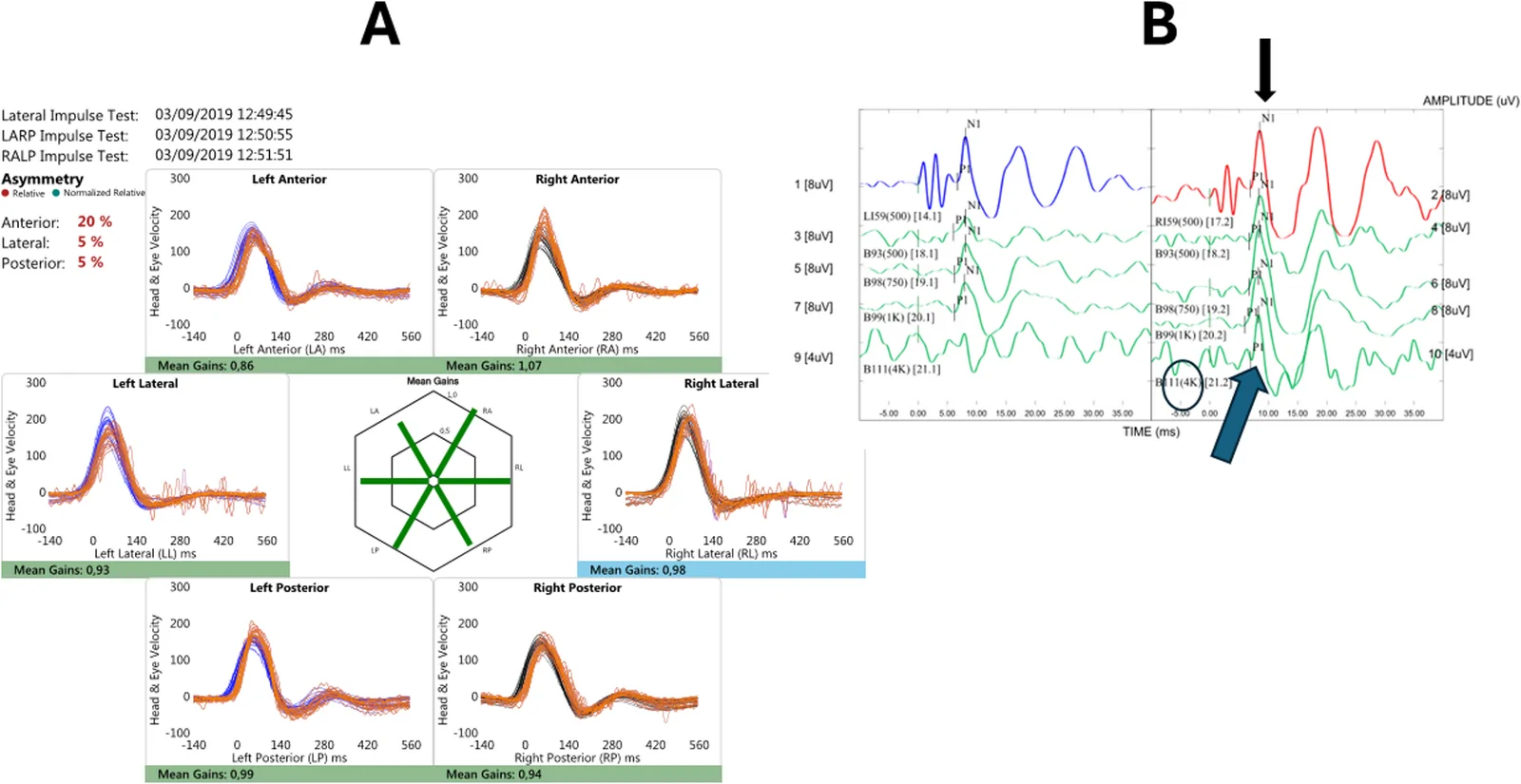
Normal vHIT with enhanced BCV- and ACS-oVEMPs in left superior semicircular canal dehiscence, vHIT and oVEMPs to bone-conducted vibration (BCV) and air-conducted sound (ACS) in a second patient with left superior semicircular canal dehiscence (SSCD). (**A**) video Head Impulse Test (vHIT) recorded at the MSA ENT Academy Center, Cassino (Italy), showing normal vestibulo-ocular reflex (VOR) gains for rapid, unpredictable head impulses in the planes of all six semicircular canals, including the left anterior (superior) canal affected by the dehiscence. No pseudo-hypofunction of the superior canal is evident, in striking contrast to the patient shown in Figure 1. (**B**) Raw traces of ocular VEMPs (oVEMPs) from the same subject. On the left are responses recorded beneath the left eye, reflecting predominantly the right (healthy) utricle; on the right are responses recorded beneath the right eye, reflecting the left (dehiscent) utricle. The top row shows BCV-oVEMPs to 500 Hz BCV delivered at Fz; the subsequent rows show ACS-oVEMPs to 500 Hz, 750 Hz, 1000 Hz, and 4000 Hz tone bursts (0 ms rise time, ms plateau). A robust n10 potential is present at 4000 Hz beneath the eye contralateral to the affected ear, together with enhanced oVEMP responses to both BCV and ACS, indicating hypersensitivity of utricular/superior canal afferents despite preserved high-acceleration canal function on vHIT. This figure illustrates that marked third-window–related oVEMP abnormalities may occur in SSCD even when vHIT gains are entirely normal.

Superior semicircular canals demonstrated the highest prevalence of dysfunction, with up to 21% in LA canals. Dysfunctions were also present in the left posterior (9%), right anterior (8%), right lateral (8%), right posterior (6%), and left lateral (2%) canals. Patients with bilateral SSCD demonstrated a significantly lower right anterior gain than those with unilateral SSCD. The LA canal exhibited an even more pronounced reduction in the same bilateral SSCD subgroup (mean 0.68 **±** 0.14), which is below the clinical hypofunction threshold. This is also confirmed by the higher prevalence of superior canal dysfunction in people with bilateral SSCD compared with unilateral cases. All included patients displayed enhanced oVEMP amplitudes at 500-Hz BCV and reliable N10 responses at 4,000 Hz. Two participants with left SCDS are described in Figs. 1 and 2; both presented enhanced BCV and ACS oVEMPs, with a pseudo-hypofunction on vHIT and a normal vHIT, respectively.

## Discussion

This study investigated high-frequency SSC function in people with SCDS using vHIT, examining the influence of unilateral versus bilateral dehiscence, and integrating these findings with high-frequency oVEMPs (including 4-kHz responses). The results show a consistent and internally coherent pattern in which high-frequency VOR alterations are selective, modulated by disease laterality, and systematically dissociated from otolith hyperresponsiveness. Across the cohort with CT-confirmed SCDS VOR gain reductions were not diffuse but selectively involved the SSCs. The left anterior semicircular canal showed the greatest impairment, with 21% of ears exhibiting abnormal gain, and both SCC displayed significantly lower VOR gains in bilateral compared with unilateral SCDS. In contrast, horizontal semicircular canal function remained essentially preserved in nearly all individuals. Posterior semicircular canal involvement, when present, was modest and likely reflected mechanical coupling effects rather than direct pathological consequences of the dehiscence. Our findings confirm that vHIT abnormalities in SCDS are canal-specific, frequency-specific, and primarily mechanically driven. The observed pattern is consistent with the expected canal-specific vulnerability inherent to SSCD biomechanics, supporting the concept that SSC hypofunction arises from altered fluid dynamics rather than global vestibular impairment or neuroepithelial injury. These results are consistent with previous observations by Mukherjee et al. [5], who reported a high prevalence of SSC hypofunction despite preserved or even exaggerated otolith-mediated responses. Similarly, our data demonstrate a pronounced vHIT–oVEMP dissociation, in which reduced high-frequency semicircular canal responses coexist with increased otolith excitability. In contrast, Tikka et al. [6] observed broader variability and weaker group differences, underscoring the need for cautious interpretation of vHIT in SCDS and reinforcing that vHIT alone should not be considered a definitive diagnostic test. Our results help reconcile these perspectives: although VOR gain reductions are not universally present, when they do occur, they follow a highly characteristic pattern localized to the dehiscent superior canal and are more pronounced in bilateral cases. A strong anatomical and biomechanical correlation for these findings is provided by Castellucci et al. [7] who demonstrated that SSC VOR gain correlates inversely with dehiscence size and is strongly influenced by dehiscence location, with arcuate-eminence defects producing the largest gain reductions. The significantly lower SSC gains in our bilateral SCDS cases are consistent with enhanced hydrodynamic shunting due to larger or more severe dehiscence. Furthermore, these alterations represent not true semicircular canal paresis but a mechanical “pseudo-hypofunction” resulting from energy dissipation through the third window and potential dynamic plugging by dural indentation [7]. Our data conform precisely to this model: VOR deficits are selective, mechanical, and frequency-specific. The question then arises: why do patients with reduced high-frequency SSC responses show enhanced utricular and superior canal responses to high-frequency acoustic and vibratory stimuli? The answer lies in the neurophysiology of the transient vestibular system. As described by Curthoys et al. [3] vHIT primarily stimulates Type I irregular afferents located at the center of the ampullary crest, which are high-frequency, jerk-sensitive receptors responsible for rapid onset VOR dynamics. These same Type I receptors mediate oVEMP responses to high-frequency stimuli, but in a different mechanical context: sound and vibration, rather than angular acceleration.

**Fig. 3:**
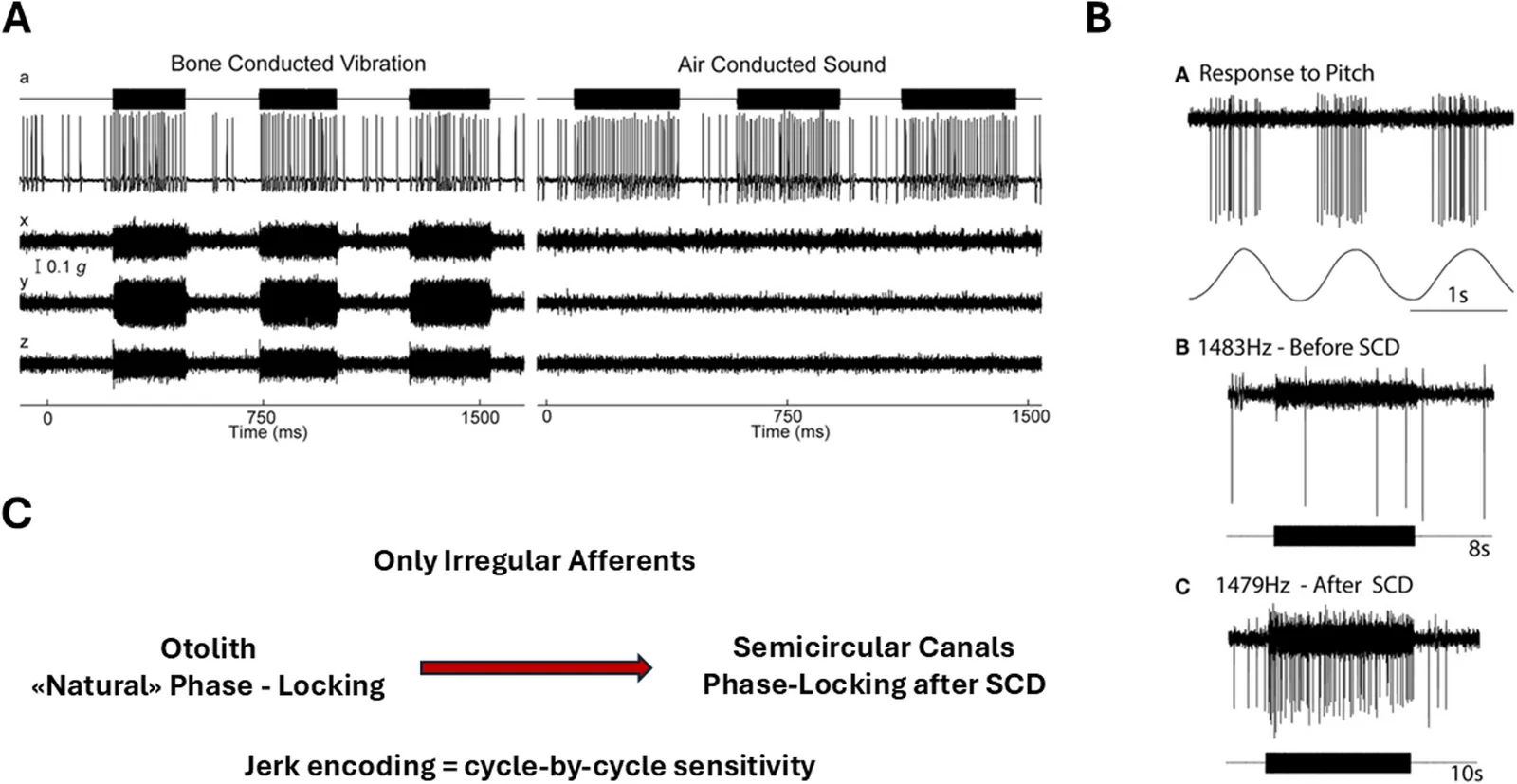
Phase locking of irregular vestibular afferents during high-frequency stimulation. (**A**) Otolith irregular afferent: Time series and circular phase histogram of a utricular irregular neuron responding to 500 Hz bone-conducted vibration (BCV). The neuron does not fire on every cycle, but each spike occurs within a narrow band of stimulus phase angles, demonstrating highly precise phase locking. Adapted from Curthoys et al. (2018) [13]. **B**) Anterior semicircular canal irregular afferent after superior canal dehiscence (SCD): Before SCD, the afferent shows no response to high-frequency air-conducted sound (ACS). After SCD, the same neuron exhibits strong phase-locked firing to each stimulus cycle, revealing restored mechanical coupling between the canal cupula and acoustic stimulation. Adapted from Curthoys et al. (2018) [14]. (**C**) Schematic comparison of the phase-locking capability of irregular utricular afferents versus irregular anterior semicircular canal afferents. Otolith afferents naturally exhibit precise cycle-by-cycle phase locking to high-frequency bone-conducted vibration, whereas canal afferents require a third-window mechanism—such as superior canal dehiscence—to display comparable phase-locked firing to air-conducted sound. Both behaviors indicate that irregular vestibular afferents function as high-speed jerk detectors with millisecond temporal precision. Adapted from Curthoys et al., 2018 (Frontiers in Neurology) [14].

Importantly, in our cohort all patients demonstrated enhanced oVEMP amplitudes at 500-Hz BCV and reliable N10 responses at 4,000 Hz. Although enhanced oVEMP responses are widely considered a hallmark of third-window physiology, we cannot completely exclude the possibility that endolymphatic hydrops may contribute to the modulation of otolith responses in some patients. Secondary hydropic changes have been reported in association with SCDS and may alter endolymphatic pressure gradients, potentially influencing the mechanical displacement of the otolithic membranes and thereby affecting VEMP amplitudes. In this context, it is conceivable that both mechanisms—third-window energy redistribution and hydropic amplification—could coexist in certain individuals. Future studies combining VEMP testing with electrocochleography or other markers of endolymphatic hydrops may help to further clarify the relative contribution of these mechanisms. This finding carries exceptional diagnostic significance. In a previous study [8], we demonstrated that the presence of a 4-kHz oVEMP N10 response has 100% sensitivity and 100% specificity for CT-confirmed SCD. All individuals with SCD showed a measurable 4-kHz N10 response, whereas none of the control participants did. As emphasized by Curthoys et al. [13] only irregular Type I utricular afferents can follow such ultra-high-frequency transient stimuli. Thus, the presence of a 4-kHz oVEMP in every patient in our cohort provides compelling evidence that Type I vestibular hair cells are intact and hyperresponsive, not impaired. This finding definitively excludes neuroepithelial hypofunction as the cause of reduced vHIT gains. Further powerful mechanistic support comes from the physiological and anatomical evidence [2] in which after even a 0.1 mm dehiscence in the superior canal, superior canal neurons that are normally silent to high-frequency sound become powerfully activated and phase-lock to 1–4 kHz acoustic stimuli as reported in Fig. 3 (adapted from Curthoys et al. [14]). Tthe same neuron is unresponsive before the dehiscence and robustly activated afterward. Crucially, both utricular and superior semicircular canal afferents project to the extraocular motor circuitry generating the oVEMP, explaining why oVEMP amplitudes are dramatically enhanced after SCD. These works collectively show that SCD modifies the mechanical input pathways such that high-frequency sound stimulates both otolith and superior canal afferents, amplifying the N10 response.

The seemingly paradoxical combination of reduced vHIT gain yet enhanced high-frequency oVEMPs is elegantly explained by the FSI model of Goyens et al. [9]. Their simulations demonstrate that early transient VOR dynamics rely on an utricle-generated endolymph vortex, which produces a characteristic asymmetric, S-shaped cupular deformation that maximally stimulates Type I receptors. In SCDS, a portion of the mechanical energy required to generate this vortex is lost through the dehiscence, blunting cupular displacement and producing vHIT hypofunction despite preserved sensory epithelium. Simultaneously, the third window enhances the transmission of acoustic and vibratory energy to both utricular and superior canal afferents, explaining the hyperfunctional oVEMP responses. Thus, SSC vHIT reduction and enhanced oVEMP amplitudes reflect two sides of the same hydrodynamic phenomenon: frequency-dependent redistribution of energy within the labyrinth.

In SCDS, however, this mechanical energy is dissipated through dehiscence, reducing vortex formation, and thus attenuating cupular displacement. The result is vHIT hypofunction despite intact sensory cells, a hallmark of mechanical pseudo-hypofunction. Simultaneously, the same low-impedance shunt greatly increases the transmission of acoustic and vibratory energy to the utricle and to superior canal afferents, explaining the enhanced oVEMPs at both 500 Hz and 4 kHz. Thus, SSC vHIT reduction and enhanced oVEMP responses are not contradictory, but rather complementary expressions of frequency-dependent hydrodynamic dissociation. Clinically, the implications are substantial. First, reduced SSC vHIT gains in SCDS should not be interpreted as evidence of permanent vestibular hypofunction. They reflect altered canal biomechanics, not irreversible canal paresis. Second, the presence of 4-kHz oVEMPs confirms utricular and canal Type I receptor integrity and provides a rapid, non-invasive screening tool for SCDS with near-perfect sensitivity and specificity. Third, the significant worsening of SSC gain in bilateral SCDS underscores the need for careful preoperative assessment to avoid iatrogenic bilateral canal dysfunction.

Taken together, our findings support a unified interpretation: SSC hypofunction on vHIT in SCDS reflects hydrodynamic energy loss rather than neuroepithelial injury, while oVEMP hyperfunction reflects enhanced energy transmission to otolith and canal afferents. This refined understanding highlights the importance of considering inner-ear fluid mechanics in the interpretation of vestibular test batteries and establishes vHIT and high-frequency oVEMPs as complementary tools for characterizing the unique biomechanical signature of SCDS.

The interpretation of reduced SSC vHIT gain in SCDS has recently been the subject of debate. An additional potential explanation for reduced SSC vHIT gains is spontaneous canal plugging, which may occur through fibrous tissue proliferation or otoconial aggregation within the canal lumen. Such structural changes could mechanically attenuate canal responses independently of hydrodynamic energy dissipation through the dehiscence. Previous imaging studies have demonstrated that autoplugging can modify vestibular responses and alter audiometric findings even in non-operated ears with SCD. Although the physiological profile observed in our cohort strongly supported an active third-window state, dedicated high-resolution imaging techniques would be required to definitively exclude structural canal obstruction in individual cases. Castellucci and colleagues [10] compared symptomatic SCD ears showing SSC hypofunction with ears that had undergone surgical plugging and concluded that reduced vHIT gain in non-operated SCDS is more plausibly explained by hydrodynamic energy dissipation at the dehiscence rather than by spontaneous canal occlusion. In contrast, Ionescu et al. [15] described cases of partial or complete spontaneous plugging verified by imaging in which vHIT gains remained normal, indicating that canal VOR transduction may be preserved even in the presence of lumen obstruction. In our cohort, however, the physiological profile was consistently that of an active third-window phenotype, characterized by enhanced 500-Hz oVEMPs, uniformly present 4-kHz N10 responses, and audiometric signs of third-window physiology. This constellation of findings makes spontaneous plugging unlikely and supports hydrodynamic energy dissipation as the most coherent explanation for the observed vHIT hypofunction. Importantly, vHIT hypofunction is not an inherent feature of all dehiscences; rather, in ears with sustained third-window hyperexcitability, reduced SSC VOR gain most plausibly reflects energy loss through the dehiscence rather than luminal occlusion. Further clarification is provided by a recent commentary [16] and which directly addresses the issues raised by Ionescu et al. [15]. This analysis reinforces that SSC hypofunction is not a universal consequence of dehiscence but becomes a consistent and clinically meaningful finding in ears that maintain an active third-window physiology. In this context, the key determinant is not simply the presence of a bony defect, but the functional state of the hydrodynamic system surrounding it.

**Fig. 4:**
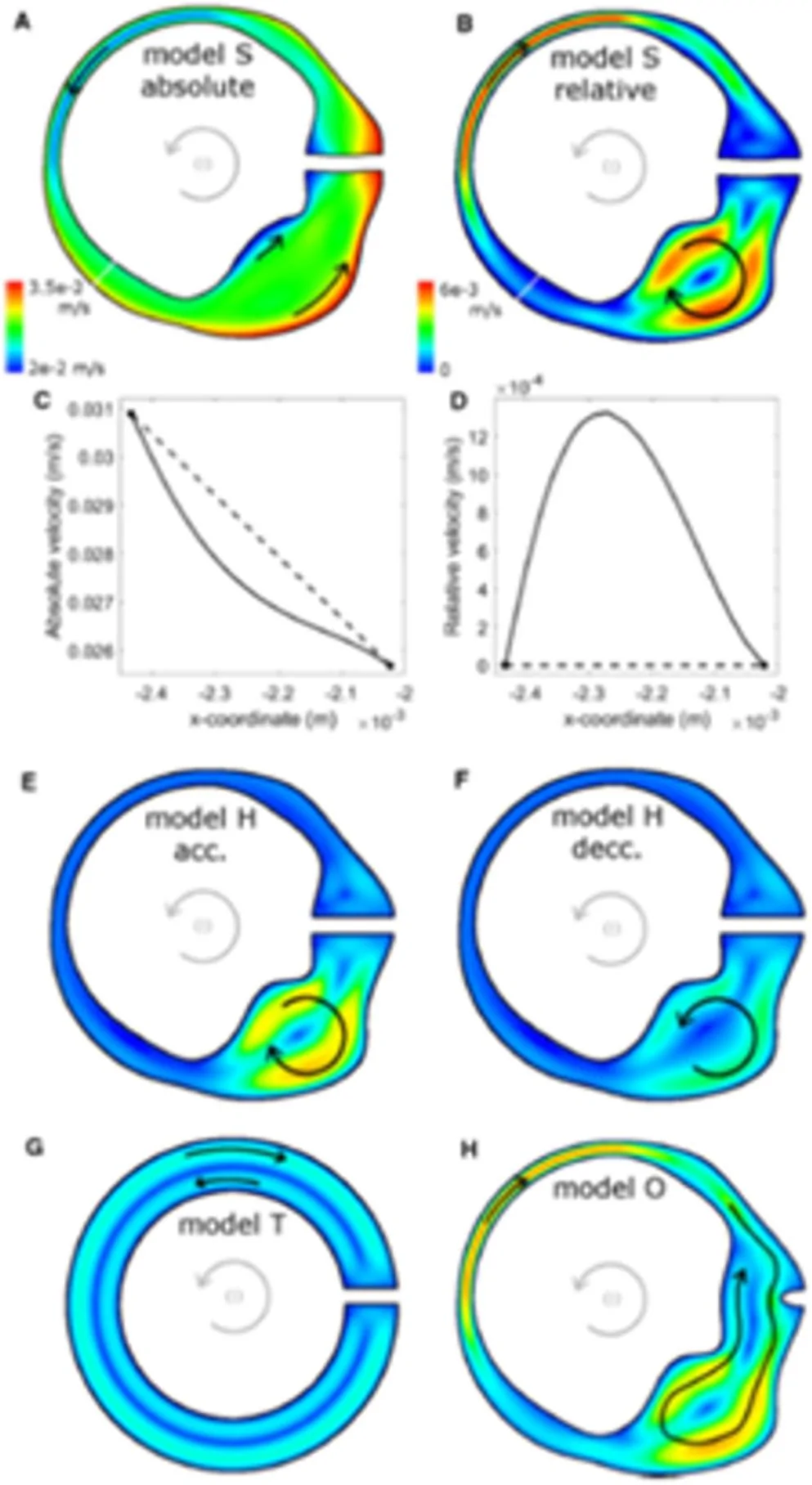
Endolymph velocity in the XY-plane of the lateral semicircular canal–utricle system during an “alarm turn” head rotation (t = 0.08 s; panel F at t = 0.16 s). (**A**, **B**) Model S: absolute (**A**) and relative (**B**) endolymph velocity fields. (**C**, **D**) Velocity magnitude along the narrow-duct cross-sections indicated by the white lines in A and B, plotted in the absolute (**C**) and relative (**D**) reference frames. (**E**, **F**) Relative velocity field in the human geometry with rigid cupula (model H) during angular acceleration (**E**) and deceleration (**F**). (**G**) Relative endolymph velocity in the torus geometry (model T). (**H**) Relative endolymph velocity for the human geometry with open ampulla (model O). The same colour scale is used for all relative-velocity maps. Grey arrows indicate the direction of head rotation; black arrows schematically indicate the direction of endolymph flow. Relative velocities are small, so individual fluid particles do not move completely from utricle to ampulla or vice versa during the manoeuvre. Re-produced from Goyens et al., Biomechanics and Modeling in Mechanobiology, 2019, with permission [9]

Crucially, Malara et al. [16] also suggested that the velocity of the head impulse is essential when evaluating SSC function. A superior canal that appears normal during low-velocity impulses may become clearly hypofunctional when vHIT is performed at appropriate high accelerations, indicating that suboptimal stimulus intensity can mask true high-frequency dysfunction. This observation has direct implications for the interpretation of apparently normal vHIT gains reported in some cases of SCDS and highlights the importance of correct test execution. Moreover, Malara et al. [16] emphasized that hydrodynamic flow dissipation through the dehiscence remains the most parsimonious explanation for SSC hypofunction in patients displaying robust third-window signs. While spontaneous plugging is possible, it is typically associated with different physiological fingerprints, such as normalization of VEMPs and resolution of air–bone gaps, patterns that were not observed in our cohort. In contrast, all our patients exhibited enhanced oVEMP amplitudes, including consistent 4-kHz N10 responses, indicating persistent third-window hyperexcitability and preserved Type I receptor function. This pattern aligns closely with the interpretation that reduced SSC gain in SCDS reflects hydrodynamic energy shunting rather than sensory failure. Within this framework, SSC vHIT hypofunction can be regarded as a physiologically reliable marker of active third-window mechanics, provided that the test is performed at appropriate velocities and interpreted in conjunction with complementary indicators such as high-frequency oVEMPs and audiometric evidence of third-window physiology. Despite extensive clinical descriptions of the vHIT–VEMP dissociation in SCDS, the biomechanical underpinnings of this phenomenon have remained largely unexplored in the clinical literature. The interpretation of the vHIT–oVEMP dissociation can be informed by the fluid–structure interaction (FSI) model proposed by Goyens et al. [9] (Fig. 4), which provides a mechanistic framework for linking endolymph flow, utricular vortex formation, and cupular deformation during high-acceleration head impulses within anatomically accurate geometry.

Although originally developed outside the otoneurological clinical literature, this model offers a critical mechanistic insight by demonstrating that early transient VOR responses depend on a utricle-driven endolymph vortex, which generates an asymmetric, S-shaped cupular deformation concentrated in the region of Type I irregular afferents.

In the presence of a dehiscence, a substantial portion of this mechanical energy is shunted away, attenuating vortex formation and consequently reducing cupular displacement.

This mechanism explains, at a fundamental physical level, the high-frequency pseudo-hypofunction observed on vHIT.

Integrating the Goyens model with our clinical data and contemporary neurophysiology therefore provides a unified biomechanical explanation for the vHIT–oVEMP dissociation in SCDS.

Rather than being ancillary, this framework enhances the internal coherence of the third-window concept and represents a central element for future physiopathological interpretations of SCDS. 

This study has several limitations. It is retrospective and single-center, potentially biasing the sample toward patients with more pronounced third-window physiology. Subgroup analyses remain constrained by sample size, especially in bilateral SCDS. Dehiscence morphology (length, precise location) was not quantified with uniform imaging protocols, preventing detailed structure–function correlations. Another important limitation is the absence of systematic low-frequency vestibular testing such as caloric irrigation or rotational chair evaluation. Because superior semicircular canal dehiscence may coexist with secondary endolymphatic hydrops, alterations in low-frequency vestibular responses could theoretically influence the interpretation of high-frequency findings. Hydropic changes may modify caloric responses or contribute to vestibular asymmetries, potentially interacting with the biomechanical effects of the third window. As low-frequency tests were not consistently available in our cohort, the present analysis focused on high-frequency vestibular responses assessed with vHIT and VEMPs. The present study was intentionally focused on dynamic vestibular testing. Future studies integrating multi-frequency vestibular evaluation may further refine the physiological understanding of SCDS. Finally, although our interpretation is grounded in robust biomechanical and neurophysiological models, direct measurements of endolymph flow or single-unit neural activity were not performed.Nonetheless, these results have important clinical implications. First, they affirm that reduced SSC vHIT gain in SCDS does not signify irreversible vestibular loss, but rather hydrodynamic pseudo-hypofunction driven by energy shunting through the dehiscence. Second, high-frequency oVEMPs, particularly the 4-kHz N10 response, provide a reliable indicator of intact Type I function, helping differentiate SCDS from true vestibular hypofunction. Third, the coexistence of SSC vHIT reduction with otolith hyperresponsiveness highlights the importance of multimodal assessment and cautions against overinterpreting vHIT findings in isolation. Finally, in bilateral SCDS, understanding the balance between hydrodynamic dysfunction and preserved sensory integrity is crucial for surgical planning to avoid generating bilateral canal impairment. The present study was intentionally designed to investigate dynamic vestibular responses, focusing specifically on the relationship between high-frequency semicircular canal performance assessed by vHIT and otolith-related responses assessed by oVEMPs in patients with SCDS. Our aim was therefore not to provide a complete multi-frequency vestibular characterization of these patients, but rather to examine whether a functional dissociation could be demonstrated within the domain of clinically relevant dynamic tests. In this context, the observed combination of reduced superior canal vHIT gain and preserved or enhanced oVEMP responses identifies a distinctive pattern consistent with differential effects of the third-window lesion on canal and otolith pathways. Low-frequency tests such as caloric irrigation or rotational chair may provide additional information on other aspects of labyrinthine physiology, but they address a different stimulus domain and were outside the specific scope of the present study. Future investigations may integrate these complementary approaches; however, their absence does not detract from the internal rationale of the current work, which was specifically centered on dynamic vestibular testing. From an interpretative perspective, our findings are best understood within a functional framework centered on third-window mechanics. The combination of reduced superior canal high-frequency response and enhanced otolith-related responses does not suggest a uniform vestibular failure, but rather a selective dissociation between dynamic canal behavior and otolith excitability. This was precisely the physiological pattern the study was designed to explore. Although additional structural or hydrodynamic factors may be discussed in broader models of SCDS, the present data support the concept that dynamic vestibular tests can reveal non-parallel behavior of different receptor systems in the same disease. In this sense, the value of the study lies not in exhaustively covering every vestibular domain, but in showing that dynamic canal and otolith tests may diverge in a clinically meaningful and mechanistically informative way. The dissociation observed in the present cohort between reduced superior canal vHIT gain and enhanced oVEMP responses may therefore reflect the redistribution of hydrodynamic energy predicted by biomechanical models of third-window lesions [9].

## Conclusion

This study demonstrates that the reduction of high-frequency SSC VOR gain observed in symptomatic SCDS represents a mechanical pseudo-hypofunction arising from hydrodynamic energy dissipation across the dehiscence, rather than a loss of sensory receptor function. All participants displayed a consistent “third-window active” phenotype, strongly indicating that the SSC hypofunction observed on vHIT does not reflect Type I receptor dysfunction. A multimodal approach incorporating high-velocity vHIT, high-frequency oVEMPs, and imaging offers the most accurate evaluation of SCDS and provides essential guidance for surgical decision-making, particularly in bilateral disease. 

## References

[1] Minor LB (2005) Clinical manifestations of superior semicircular canal dehiscence. Laryngoscope 115(10):1717–1727 10.1097/01.mlg.0000178324.55729.b7 16222184

[2] Curthoys IS, Manzari L (2020) A simple specific functional test for SCD: VEMPs to high frequency (4,000 Hz) stimuli—their origin and explanation. Front Neurol 11:612075 10.3389/fneur.2020.612075 PMC7720427 33329372

[3] Curthoys IS, Manzari L, Rey-Martinez J, Dlugaiczyk J, Burgess AM (2021) Enhanced eye velocity in head impulse testing—a possible indicator of endolymphatic hydrops. Front Surg 8:666390 10.3389/fsurg.2021.666390 PMC8138434 34026816

[4] Fernández C, Baird RA, Goldberg JM (1988) The vestibular nerve of the chinchilla. I. Peripheral innervation patterns in the horizontal and superior semicircular canals. J Neurophysiol 60(1):167–181 10.1152/jn.1988.60.1.167 3404215

[5] Mukherjee P, Chiarovano E, Cheng K, Manzari L, McGarvie LA, MacDougall HG (2021) Video-head impulse test in superior canal dehiscence. Acta Otolaryngol 141(5):471–475 10.1080/00016489.2021.1884287 33641579

[6] Tikka T, Mohd Slim MA, Gaggini M, Kontorinis G (2021) Video head impulse test (vHIT) findings in patients with superior semicircular canal dehiscence: A case–control study. J Int Adv Otol 17(2):103–108 10.5152/JIAO.2021.8572 PMC9450086 33893778

[7] Castellucci A, Piras G, Del Vecchio V, Crocetta FM, Maiolo V, Ferri GG, Brandolini C (2021) The effect of superior canal dehiscence size and location on audiometric measurements, vestibular-evoked myogenic potentials and video-head impulse testing. Eur Arch Otorhinolaryngol 278(4):997–1015 10.1007/s00405-020-06169-3 32592013

[8] Manzari L, Burgess AM, McGarvie LA, Curthoys IS (2013) An indicator of probable semicircular canal dehiscence: ocular vestibular evoked myogenic potentials to high frequencies. Otolaryngology–Head Neck Surg 149(1):142–145 10.1177/0194599813489494 23674567

[9] Goyens J, Pourquie MJBM, Poelma C, Westerweel J (2019) Asymmetric cupula displacement due to endolymph vortex in the human semicircular canal. Biomech Model Mechanobiol 18(6):1577–1590 10.1007/s10237-019-01160-2 31069593

[10] Castellucci A, Malara P, Martellucci S, Alfarghal M, Brandolini C, Piras G, Ghidini A (2023) Impaired Vestibulo-Ocular Reflex on Video Head Impulse Test in Superior Canal Dehiscence:Spontaneous Plugging or Endolymphatic Flow Dissipation? Audiol Res 13(5):802–820 10.3390/audiolres13050071 PMC10604197 37887852

[11] von Elm E, Altman DG, Egger M, Pocock SJ, Gøtzsche PC, Vandenbroucke JP, STROBE Initiative (2008) The Strengthening the Reporting of Observational Studies in Epidemiology (STROBE) statement: guidelines for reporting observational studies. J Clin Epidemiol 61(4):344–349. 10.1016/j.jclinepi.2007.11.008 18313558

[12] Manzari L, Orejel Bustos AS, Princi AA, Tramontano M (2022) Video Suppression Head Impulses and Head Impulses Paradigms in Patients with Vestibular Neuritis: A Comparative Study. Healthc Basel Switz 10(10):1926. 10.3390/healthcare10101926 PMC9601449 36292373

[13] Curthoys IS, Burgess AM, Goonetilleke SC (2019) Phase-locking of irregular guinea pig primary vestibular afferents to high-frequency (> 250 Hz) sound and vibration. Hear Res 373:59–70 10.1016/j.heares.2018.12.009 30599427

[14] Curthoys IS, Grant JW, Burgess AM, Pastras CJ, Brown DJ, Manzari L (2018) Otolithic Receptor Mechanisms for Vestibular-Evoked Myogenic Potentials: A Review. Front Neurol 9:366. 10.3389/fneur.2018.00366 PMC5980960 29887827

[15] Ionescu EC, Mustea E, Reynard P, Thai-Van H (2024) Comment on Castellucci Impaired Vestibulo-Ocular Reflex on Video Head Impulse Test in Superior Canal Dehiscence:Spontaneous Plugging or Endolymphatic Flow Dissipation? Audiol. Res. 2023, 13, 802–820. Audiology Research, 14(5), 857–860 10.3390/audiolres14050072 PMC11504092 39452464

[16] Malara P, Martellucci S, Castellucci A (2025) Defining Potential Pathomechanisms Behind an Impaired Canal Function at the Video-Head Impulse Test in Canal Dehiscence. Reply to Ionescu et al. Comment on Castellucci et al. Impaired Vestibulo-Ocular Reflex on Video Head Impulse Test in Superior Canal Dehiscence:Spontaneous Plugging or Endolymphatic Flow Dissipation? Audiol. Res. 2023, 13, 802–820. Audiol Res 15(2):32 10.3390/audiolres15020032 PMC11932197 40126280

